# Liquid Silicone Rubber Injection Molding: A Review of Material–Process–Quality Relationships

**DOI:** 10.3390/polym18212623

**Published:** 2026-10-23

**Authors:** Weimin Yang, Pengfei Zhang, Guannan Ren, Zhongjie Li, Pengcheng Xie, Kaifang Dang

**Affiliations:** College of Mechanical and Electrical Engineering, Beijing University of Chemical Technology, Beijing 100029, China; 1990500018@buct.edu.cn (W.Y.); 2025200728@buct.edu.cn (P.Z.); 2024410030@buct.edu.cn (G.R.); leezhjie@buct.edu.cn (Z.L.)

**Keywords:** liquid silicone rubber, injection molding, curing kinetics, process monitoring, molding equipment, part quality

## Abstract

Liquid silicone rubber (LSR) injection molding produces elastomeric components with complex geometries and demanding functional requirements through coupled flow, heat transfer, and crosslinking. This review examines how formulation, processing conditions, and equipment operation jointly affect part quality. Literature was identified through Web of Science and Google Scholar searches and citation tracing, with no lower publication-year limit and a search cutoff of September 2026. Rheological behavior and curing kinetics underpin the discussion of material delivery, cavity filling, and demolding, alongside equipment technologies for metering, injection, thermal management, and process monitoring. Available evidence links processing history to shrinkage, surface replication, mechanical properties, and defects. Biomedical, optical, multi-material, and soft robotic applications illustrate how functional requirements guide manufacturing choices. Processing conditions influence multiple quality outcomes simultaneously, requiring their selection to account for formulation, geometry, and intended function. A key gap remains in linking process signals, independently characterized material states, and effective control actions across formulations and molds. Future priorities include material-state-based control, thermal efficiency, silicone reuse and recovery, and hybrid manufacturing. By relating equipment functions to material responses and measurable quality outcomes, this review supports application-specific selection of processing and evaluation methods.

## 1. Introduction

Liquid silicone rubber (LSR) combines thermal stability with elastomeric flexibility, making it suitable for components with demanding performance requirements [1]. As embodied intelligence and human–machine interaction technologies advance, soft robots and wearable devices place increasing demands on compliant components that can accommodate deformation and contact while incorporating complex geometries. Together with the demand for flexible materials in healthcare devices, these requirements have broadened the applications of silicone-based materials [2,3,4]. Translating material capabilities into device performance requires not only suitable formulations but also manufacturing processes that consistently achieve the required geometry and properties. Injection molding delivers the reactive LSR compound into a heated mold for filling and curing. Its suitability for automation and repeatable production makes it an important manufacturing route for complex LSR parts [5,6,7].

However, automation and repeatable production do not by themselves ensure consistent part quality; control of the evolving material state during filling and curing is also essential. The relatively low viscosity and shear-thinning behavior of uncured LSR facilitate flow into complex cavities, but progressive network formation in the heated mold restricts that flow. Filling is therefore coupled with heat transfer and curing rather than occurring as an independent material-delivery step. These interactions are governed jointly by rheology, reaction kinetics, injection conditions, and mold design [8,9]. Changes in injection conditions alter the flow and thermal histories experienced by the material, while curing in turn affects subsequent filling. These process differences influence network formation, residual stress, and surface replication, with consequences for dimensional accuracy and mechanical properties.

In production, the flow and thermal conditions experienced by the material are largely determined by the operation of metering, injection, and temperature-control equipment. The Si–H/vinyl ratio and filler characteristics influence rheological and curing behavior, while equipment regulates the material response during the molding cycle through component delivery, injection, and heat transfer. Accurate dosing and adequate mixing, for example, help maintain the specified component ratio and mixture homogeneity; cooled delivery systems limit premature crosslinking before mold entry; and mold heating promotes network formation within the cavity. The effects of equipment operation are therefore closely linked to material characteristics: the same injection or temperature-control settings may produce different filling and curing histories across formulations and part geometries.

Existing reviews have discussed silicone rubber from complementary perspectives. Xing et al. focused on composition–property relationships in high-temperature-resistant addition-cure LSR [1], while Huang and Chen linked silicone rubber formulations and manufacturing methods to industrial applications [6]. Bont et al. addressed LSR injection-molding stages, process variables, material models, and simulation, including process feedback [10]. Table 1 compares the scope and emphasis of these reviews with those of the present review. Building on these contributions, the present review organizes evidence around how equipment operation influences material responses and how these changes are reflected in part quality. It distinguishes measured process responses, inferred material states, and independently assessed part quality, providing a basis for evaluating monitoring and control approaches in relation to application-specific manufacturing requirements.

Literature for this review was identified primarily through keyword searches in Web of Science and Google Scholar, supplemented by citation tracing. The search was last updated in September 2026. Literature was selected based on its relevance to material formulation, processing behavior, equipment operation, and part quality in LSR injection molding. Peer-reviewed studies on LSR constitute the primary evidence base, covering rheology and curing, material delivery and cavity filling, thermal management, process monitoring, and application-specific performance. Selected patents, technical reports, and industrial publications provide additional information on equipment configurations and manufacturing practice. Studies on other silicone systems and thermoplastic injection molding are included when they help explain mechanisms or methods relevant to LSR processing, with their material systems and processing conditions specified in the discussion. The literature is organized thematically to compare how formulations and operating conditions influence material responses and manufacturing outcomes.

To develop this discussion, the review first introduces the material characteristics of LSR and its flow and curing behavior, followed by an examination of the roles of metering, injection, cold-runner systems, and mold temperature control in molding. The effects of processing conditions on part quality are then discussed in terms of dimensional shrinkage, surface replication, mechanical properties, and defect formation. Medical, optical, and multi-material applications illustrate how functional requirements guide process selection. Finally, current limitations and future directions are considered in material-state sensing and control, thermal management, resource recovery and reuse, and hybrid manufacturing.

## 2. Material Characteristics Governing LSR Injection Molding

### 2.1. Composition, Crosslinking Chemistry, and Formulation

The processing behavior of LSR originates in its formulation and crosslinking chemistry. Commercial injection-grade LSR typically consists of a two-component addition-cure system containing vinyl-functional polydimethylsiloxane (PDMS), a hydrogen-containing siloxane crosslinker, a platinum catalyst, an inhibitor, and reinforcing silica [11,12,13,14]. The siloxane backbone and reactive functional groups underpin the material’s flexibility and capacity to form a crosslinked network [15,16]. For processing, the two components are stored separately and accurately metered and mixed before entering the cooled injection unit [10]. This arrangement allows the reactive compound to be prepared shortly before molding, while inhibition and temperature control preserve its flowability during delivery to the heated mold.

Once heated, the compound undergoes hydrosilylation between Si–H and vinyl groups, progressively forming a three-dimensional network [13,14]. The structure of this network connects formulation to mechanical performance through crosslink density, molecular weight between crosslinks, and polymer–filler interactions [17,18]. The relative concentrations of Si–H and vinyl groups influence network development, whereas catalyst activity and inhibition govern the rate of reaction and the time available for processing [19,20]. Rheological studies of two-component addition-cure silicone rubber trace this transformation through the evolving material response during curing [21]. Formulation therefore influences both the properties of the cured elastomer and the period over which the compound remains sufficiently fluid for molding.

Alongside the reactive components, fillers modify both material performance and the conditions required for processing. Functionalized graphene oxide illustrates their use in tailoring the thermal and mechanical properties of LSR [22], while studies of surface-modified fillers show that reinforcement and curing depend on the chemistry of the filler–polymer interface. Vinyl-functionalized silica improved dispersion and reduced uncured viscosity relative to unmodified silica, while providing reactive sites for integration into the cured network [23]. By contrast, cellulose nanocrystals modified with 3-aminopropyltriethoxysilane improved mechanical performance but increased the curing temperature, which the authors attributed to inhibition of the platinum catalyst by the surface modifier [24]. These contrasting effects show why improved interfacial compatibility does not necessarily imply faster curing. Filler geometry also affects processing behavior and heat transport, as demonstrated by the comparison of thermally conductive fillers in silicone-rubber composites [25]. Filler selection therefore involves coordinating surface chemistry, dispersion, and geometry with the flowability and curing response needed to manufacture the part.

### 2.2. Material Behaviors Governing Injection Molding

Before substantial crosslinking occurs, filler-related physical structure also contributes to the rheological response, distinct from the chemical network formed during curing [26]. Surface modification can reduce this resistance to flow while retaining reinforcement, as demonstrated for modified silica in LSR [8]. The effect is therefore not described by filler content alone. In a study of vinyl-functionalized silica, a power-law description was used to characterize the shear response through the consistency coefficient and flow index [23]. Such parameters summarize how a particular compound flows over the measured shear-rate range, providing a link between formulation and the material description used in filling analysis. In practice, comparing formulations under relevant shear and temperature conditions helps determine the pressure required for delivery and the ability to reproduce fine cavity features.

Once the mixture enters the heated mold, temperature and reaction progress jointly alter its flow behavior. In the early heating stage, viscosity may decrease to facilitate filling; at the same time, the accelerating hydrosilylation reaction drives crosslinked network formation and restricts material flow. Batch, Macosko and Kemp coupled curing kinetics with heat transfer in LSR injection molding, demonstrating that gel time depends strongly on formulation and mold temperature [27]. Accordingly, the available filling window arises from the combined effects of initial flow resistance, temperature rise profile and reaction rate: while crosslinking proceeds, the mixture must retain sufficient flowability to fully fill the cavity and its local features. Formulation factors discussed in Section 2.1 affect distinct aspects of this balance. The Si–H/vinyl ratio governs network formation, catalyst activity and inhibition modulate its evolution, and filler characteristics modify flow resistance and may further influence curing and heat transfer. As curing proceeds, the composition of reactive components together with local temperature histories shape the developing network, including crosslink density and its spatial variation. Therefore, formulation and processing jointly determine the time available for filling and the network structure that governs the mechanical response of molded parts.

Describing these behaviors in molding simulations requires material data appropriate to the prediction target. Predicting filling and pressure requires a viscosity model that captures the effects of shear rate, temperature, and cure evolution. Predictions of temperature and cure distributions additionally require density, specific heat capacity, thermal conductivity, and reaction kinetics, together with reaction enthalpy when the model accounts for curing heat [10,28]. Predictions of dimensional change and residual stress further rely on pressure–specific volume–temperature behavior and an appropriate description of the material’s evolving mechanical response [10]. Available material datasets vary in the completeness of these inputs. Bont et al. noted incomplete pressure–specific volume–temperature and mechanical-model data in some commercial LSR databases, together with limitations in curing measurements that can necessitate extrapolation to molding conditions [10]. Azevedo et al. further demonstrated that characterization methods affect viscosity, specific heat capacity, and kinetic inputs, with different consequences for predicted pressure and cure evolution [28]. Material characterization should therefore provide both the parameters required by the model and the conditions under which the data were measured, so that predictions of filling, curing, and final dimensions are based on a consistent material description.

## 3. Processing Science of LSR Injection Molding

### 3.1. Metering, Mixing, and Material Delivery

A suitable formulation provides the foundation for LSR molding, but consistent processing also depends on how the compound is prepared and delivered. Components A and B are metered at the specified ratio and mixed before entering the cooled injection unit, with an additional feed stream used for pigments or other low-content additives when required [29]. The behavior of the resulting mixture depends on both its composition and rheological properties. Studies of LSR containing recycled particles show that changes in viscosity affect subsequent injection-molding behavior [30], connecting feed preparation with the flow response during injection.

Variations introduced at this stage can persist through curing and into the final part. Studies of non-stoichiometrically crosslinked PDMS relate composition to gel-point viscoelasticity [31], while Delebecq and Ganachaud showed that excess Si–H groups can alter the average molar mass between crosslinks and generate additional highly crosslinked domains [13]. These findings help explain why mixing ratio affects more than the consistency of material delivery. In optical LSR, deviations from the nominal A/B ratio changed crosslinking temperature, reaction enthalpy, viscosity, and cavity pressure, together with shrinkage, density, hardness, tensile strength, and elongation at break [32]. Composition control therefore influences both the response of the compound during molding and the properties of the network formed during curing.

In practice, feed preparation also establishes the gas content and thermal history of the compound. Air introduced during handling or mixing can enter the injection unit with the material, making degassing useful where appreciable air entrainment occurs, particularly in premixed or laboratory-prepared formulations. After mixing, cooling and residence-time control limit premature crosslinking during delivery. Together, these operations supply successive molding cycles with material of consistent composition, homogeneity, gas content, and reactive state.

### 3.2. Mold Filling and Injection Dynamics

Once the prepared compound enters the mold, injection conditions determine how it advances through the runner and cavity while being heated. Injection speed affects flow-front progression, shear conditions, pressure, and the time available for heat transfer during filling. Choi and Kim used mold-flow simulation to evaluate curing imbalance associated with runner design and injection speed, and measured the tear strength of the molded specimens [33]. The simulations provided a process-based interpretation of the experimentally observed mechanical differences. Selecting injection speed therefore involves coordinating material flow with the geometry and thermal conditions of the mold.

For microstructured parts, this coordination must extend from the main cavity to individual surface features. Material must penetrate these features while sufficient flowability remains. Weißer et al. linked LSR viscosity and processing conditions to microstructure formation [34], showing how the rheological response contributes to local replication. Overall cavity filling and feature replication thus describe different scales of the same process: the former tracks the advance of the bulk material, while the latter depends on local flow into small recesses as curing develops.

Coupled thermal, rheological, and curing simulations provide a way to examine these effects together [35]. Their application extends to multi-material molding, as demonstrated by the experimental and numerical investigation of polyamide 66/LSR bi-injection molding by Sahli et al. [36]. Within such simulations, material characterization directly influences the predicted response. Using viscosity datasets obtained through different characterization methods, Azevedo et al. predicted similar flow-front profiles but substantially different filling pressures [28]. Flow-front position and pressure history therefore provide complementary information: one describes filling progression, while the other helps assess whether the material model captures the resistance to flow.

### 3.3. Heat Transfer and Curing Kinetics

As filling proceeds, heat transferred from the mold progressively changes the compound from a flowable material into a load-bearing elastomer. Platinum-catalyzed hydrosilylation is strongly temperature-dependent, so the extent of curing develops according to the temperature history experienced by the material [37]. Curing-kinetics and reaction-modeling studies, including those by Hong and Lee [38], describe this dependence. Applying these descriptions to molding requires consideration of heat transfer within the part, because material near the mold wall and material farther from it can follow different temperature histories.

Gelation marks a transition in this developing network. Classical studies characterize it through the linear viscoelastic response [39], and Harkous et al. examined LSR gel-point determination using thermal and rheological approaches [40]. Beyond gelation, further network development gives the part the mechanical integrity needed for demolding. Weißer et al. calculated temperature and crosslinking evolution for different mold temperatures and part thicknesses [41], extending the analysis from gelation to predicted spatial variations in curing within a molded part. Gel-time measurements and demolding-time predictions consequently describe successive stages of the same transformation.

To calculate this evolution, kinetic approaches express reaction progress in different ways [42]. Phenomenological models such as the Kamal–Sourour model combine nth-order and autocatalytic terms to describe the reaction rate [43], whereas isoconversional methods estimate apparent activation energy as a function of conversion [44]. Ke et al. applied model-free kinetic analysis to addition-cure LSR [45]. These approaches offer complementary descriptions: fitted rate equations support process calculations, while conversion-dependent activation energies help characterize changes in kinetic behavior as curing progresses.

For filled formulations, applying these kinetic descriptions requires separating reaction progress from changes in the physical filler structure. Azevedo et al. showed that calorimetric and rheological measurements of the same commercial LSR supported different kinetic descriptions; they also associated continued torque growth during rheological testing with silica filler interactions [28]. Calorimetry and rheology therefore provide complementary information on reaction heat and mechanical development, consistent with comparisons of thermal and rheological approaches to LSR curing [40]. Filler effects can be incorporated into process models through formulation-specific viscosity, kinetic, and thermal-property data. This distinguishes a change in chemical reactivity, such as the catalyst inhibition reported for modified cellulose nanocrystals [24], from a change in the rate at which heat reaches the material. Together, these descriptions connect filler selection with the time available for filling and the curing time needed before demolding.

**Figure 1:**
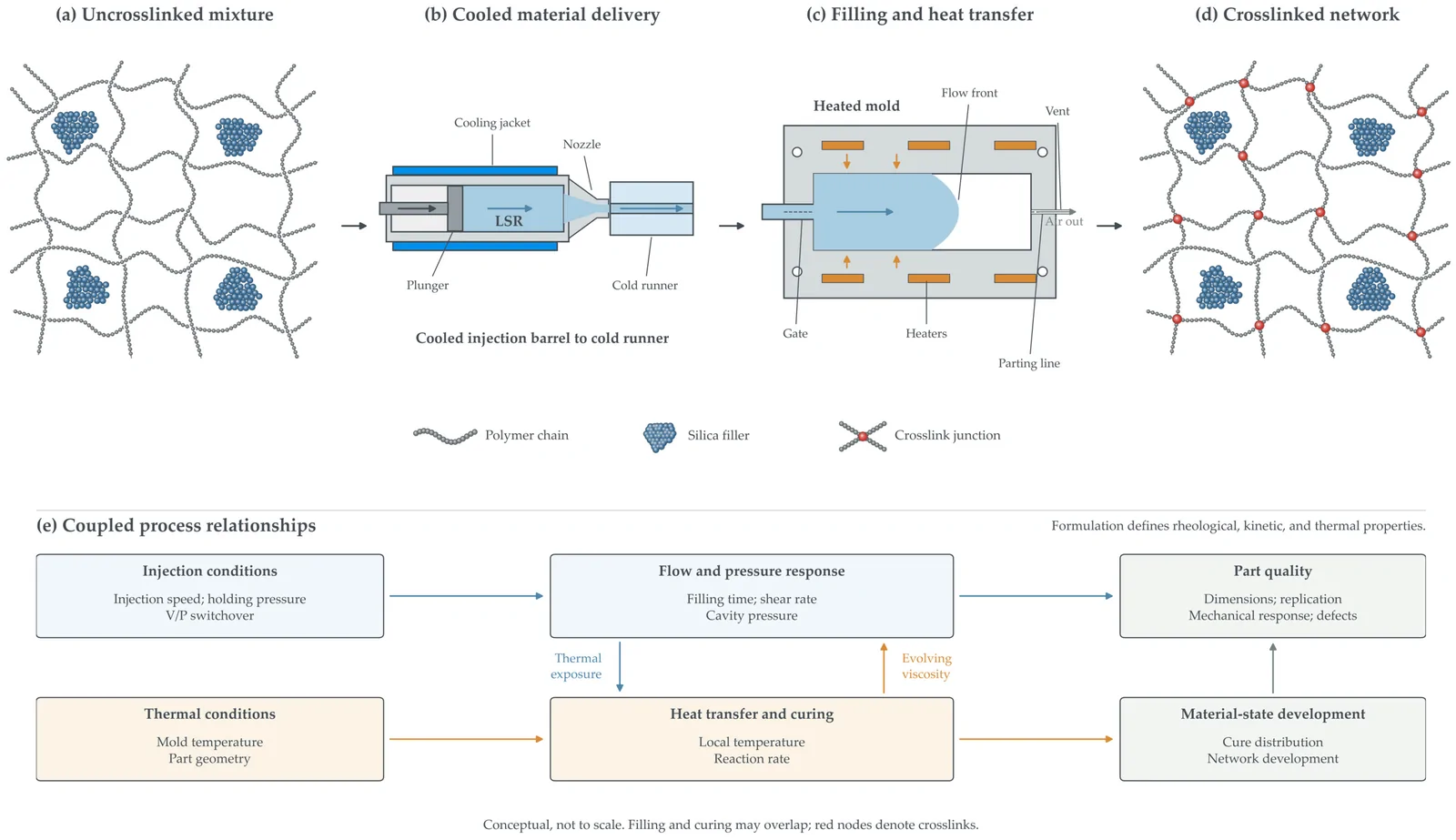
Processing stages, material-state evolution, and coupled process relationships in LSR injection molding. (**a**) Uncrosslinked polymer chains and silica filler; (**b**) material delivery through a cooled injection unit and cold runner; (**c**) cavity filling, heat transfer from the heated mold, and air displacement through the vent; (**d**) formation of a crosslinked polymer network; and (**e**) a unified process map linking injection and thermal conditions with flow, pressure, curing, material-state development, and part quality. The opposing arrows between flow and curing represent the effects of thermal exposure and evolving viscosity. V/P denotes velocity-to-pressure switchover. Filling and curing may overlap. Structures and geometries are schematic and not to scale; red nodes denote crosslinks, whereas unmarked chain overlaps do not.

Coupling reaction kinetics with transient heat transfer then allows temperature and curing distributions to be predicted throughout the part [46]. Weißer et al. compared simulated temperatures with in-cavity measurements and used the coupled model to calculate crosslinking distributions and estimate curing times for different mold temperatures and part thicknesses [41]. The calculations indicated a relatively small contribution of reaction heat under the conditions studied, while resolving spatial differences in curing. The measured temperatures support the thermal description, and the predicted crosslinking distributions provide a basis for estimating the time required before demolding. Figure 1a–d illustrates this material-state evolution through cooled delivery, cavity filling and heat transfer, and the formation of a crosslinked network.

### 3.4. Process–Structure–Quality Relationships

The flow and curing histories established during molding are reflected in the dimensions and properties of the finished part. Park et al. showed experimentally that changes in injection-molding conditions altered both cavity-pressure histories and mechanical strength [47]. The pressure records and mechanical tests establish the measured process and property responses, respectively. Network development provides a mechanistic framework for interpreting their relationship, with structural characterization needed to identify the network changes involved.

Directional shrinkage illustrates how this processing history can become embedded in the part. Bont and Johnston measured anisotropic shrinkage under different molding conditions, with flow-to-transverse shrinkage ratios ranging from below 0.92 to above 1.30 [48]. Combining dimensional measurements, dynamic mechanical analysis, and simulated curing behavior, they proposed that flow-induced orientation interacting with curing explains the observed anisotropy. The variation in shrinkage ratio indicates that dimensional compensation needs to account for flow direction and processing conditions, rather than relying on a single uniform correction.

At the surface, a related interaction between flow and curing governs replication. Studies of microstructured LSR molding connect viscosity and processing conditions with the ability to reproduce fine features [34]. For optical components, the resulting surface geometry must also be considered alongside dimensional change and internal stress. Chang et al. combined sequential approximation optimization with radial-basis-function models to identify processing conditions targeting residual stress and volumetric shrinkage in silicone lenses [49]. This model-based selection illustrates how multiple quality objectives can be considered together when choosing candidate processing conditions.

Figure 1e brings these relationships together by linking injection and thermal conditions to material-state development and part quality. Injection speed, holding pressure, and V/P switchover shape the flow and pressure histories, while mold temperature and part geometry influence heat transfer to the compound. These pathways interact: flow determines the thermal exposure experienced during filling, while temperature and advancing crosslinking modify viscosity and the resulting pressure response [10,27,28,33]. Quality therefore develops through both cavity filling and subsequent material-state evolution. Local filling affects feature replication and defect formation, while the distribution of curing and network development contributes to dimensional and mechanical responses [34,41,47,48]. This coupled view provides a basis for examining how the equipment discussed in the following section establishes and coordinates the relevant processing conditions.

## 4. Equipment Technologies for Precision Manufacturing

### 4.1. Metering and Mixing

In production, LSR molding equipment must control material delivery and temperature throughout preparation, injection, and curing. At the beginning of this sequence, the metering and mixing system controls the composition and homogeneity of the material supplied to the injection unit. In conventional A/B feeding, the components are delivered from separate containers at the specified ratio, with an additional pigment stream where required [50]. The streams pass through a static mixer, whose fixed elements repeatedly divide, redirect, and recombine the material [51,52]. Metering establishes the component proportions, while mixing distributes them throughout the compound. Dynamic mixers provide additional mixing intensity or conveying capability where needed. The feeding-unit design described by Hong and Wang illustrates how these functions can be integrated with an LSR injection-molding machine [53].

Greater formulation flexibility can be introduced by adjusting individual constituents during feeding. A patented system independently meters catalysts, crosslinkers, inhibitors, and functional additives shortly before molding [54]. This arrangement extends equipment-level adjustment beyond the conventional A/B ratio, allowing the supplied formulation to be modified through several feed streams. Its operation depends on coordinating different delivery rates and distributing low-content constituents uniformly, linking formulation flexibility directly to the precision of the dosing and mixing system.

### 4.2. Injection Units

**Figure 2:**
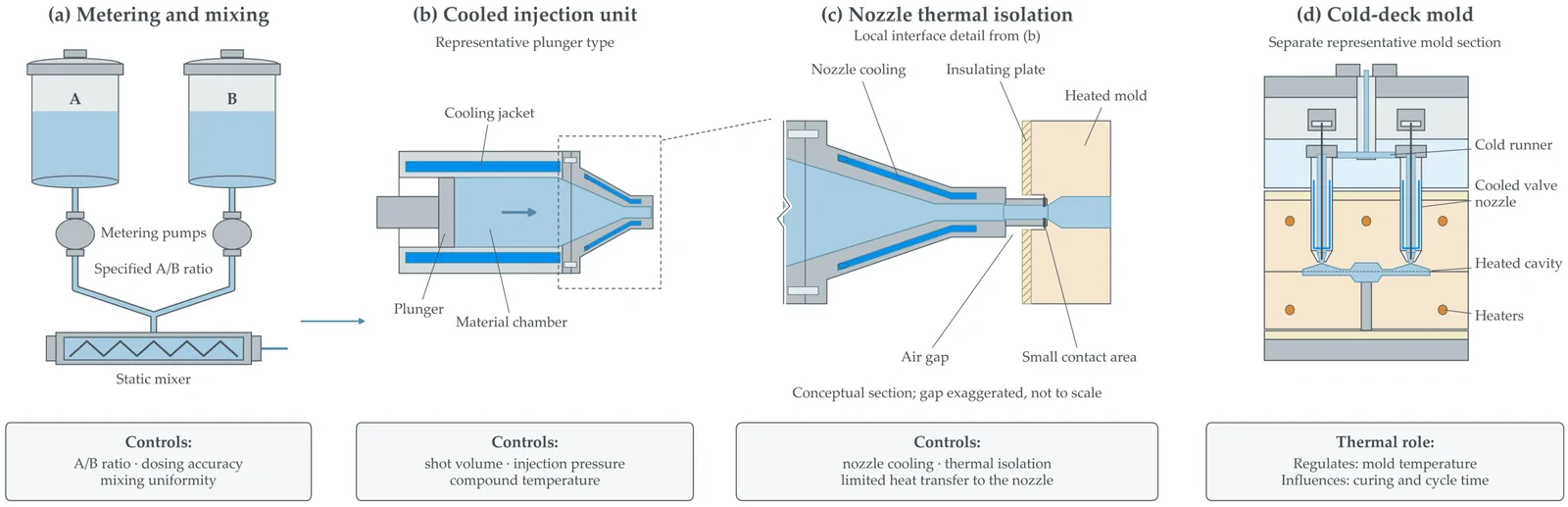
Equipment configuration and thermal management in LSR injection molding. (**a**) Component metering and mixing; (**b**) a representative cooled plunger injection unit; (**c**) an enlarged conceptual view of the nozzle–mold interface, showing nozzle cooling, an air gap, an insulating plate, and limited contact area; and (**d**) a separate representative cold-deck mold section with cooled valve nozzles and heated cavities. Panel (**c**) details the interface indicated in panel (**b**), whereas panel (**d**) presents a separate mold configuration. Material passages and cooling channels are distinguished by color. Geometries and gaps are schematic and not to scale.

After mixing, the injection unit delivers a defined shot while maintaining the compound in a processable state. LSR equipment combines a cooled barrel with a heated mold, limiting premature crosslinking during delivery and promoting curing after cavity entry [50,55]. In screw-based systems, the screw meters and injects the compound, while temperature control of the metering cylinder and mixer helps maintain flowability [50]. Non-return and shut-off elements accommodate the low-viscosity feedstock and control backflow and material discharge, contributing to consistent shot delivery [10]. In a plunger-type unit, an advancing plunger displaces the compound from a cooled material chamber toward the nozzle. Figure 2b uses this configuration to illustrate the material chamber, injection motion, and cooling path.

This operation also depends on coordination with the upstream feeding system. An ENGEL technical report describes an industrial LSR cell in which Open Platform Communications Unified Architecture (OPC UA) communication following Euromap 82.3 connects the dosing unit and injection-molding machine [56]. Such communication supports coordinated supply and injection, while the compound’s rheology determines its response to the selected settings. Heienbrock et al. showed that incorporating recycled particles changes LSR viscosity and subsequent injection-molding behavior [30]. Equipment coordination and material response therefore need to be considered together when establishing injection conditions for a modified feedstock.

At a controlled compound temperature, shot-volume and pressure repeatability provide practical measures of injection-unit performance. Barrel cooling, the injection mechanism, nozzle geometry, and shut-off behavior all contribute to these responses. Evaluating them under consistent material and shot conditions helps identify how effectively the unit reproduces the delivery conditions required by the mold.

### 4.3. Runner and Mold Technologies

The runner system extends controlled material delivery from the injection unit to the cavity entrances. In industrial LSR molding, cold runners combine cooling and thermal isolation to retain uncured compound between cycles, while needle-valve nozzles regulate delivery to individual cavities. Patent examples describe cooling channels around injection nozzles and insulation between the cold-runner plate and heated mold region [57], together with assemblies incorporating insulation plates, cold nozzles, and valve-needle mechanisms [58]. These features operate as an integrated thermal and flow-control system: runner geometry and nozzle placement distribute the compound, while cooling and insulation limit heat transfer from the mold to the upstream material.

Once the compound enters the cavity, displaced air must escape through paths that also restrict material leakage. Venting and sealing are therefore closely connected. Insufficient air removal can lead to trapped gas and incomplete local replication, whereas excessive vent clearance can permit flash; vacuum assistance and vent geometry are consequently selected together with the filling and curing conditions [10]. Kang et al. varied mold temperature, injection speed, injection pressure, and vacuum conditions when examining the replication of electrical-discharge-machined (EDM) textures, obtaining a maximum water contact angle of approximately 148° within the investigated range [59]. Their results connect surface function with the combined effects of filling conditions and air removal during texture replication, extending quality assessment beyond completion of the main cavity.

The tooling route determines how these flow and thermal functions can be implemented. Kuo and Lin investigated rapid tooling for LSR injection molding, with related work examining epoxy-based tools containing energy-efficient vulcanization channels [60,61]. Villani et al. explored tooling fabricated by fused deposition modeling under room-temperature, low-pressure, manual-injection conditions [62]. These studies address different operating regimes, from thermally managed tooling to low-pressure fabrication. Tool selection accordingly depends on the pressure, temperature, replication accuracy, and service life required by the intended process.

Within the assembled mold, these requirements converge at the runner–cavity interface. The runner must remain sufficiently cool to preserve flowability, the cavity must receive enough heat for curing, and the parting and venting surfaces must release air while containing the compound. Mold design brings these functions together through the placement of passages, thermal barriers, and sealing surfaces.

### 4.4. Thermal Control

Maintaining this separation between the cooled delivery system and heated cavity requires control over both heat supply and heat-transfer paths. Because crosslinking in two-component addition-cure LSR is temperature-dependent [21,63], the location of heaters or thermal-fluid channels relative to the part influences the development of curing across the cavity. Embedded electrical heaters and circulating thermal fluids are common ways of supplying heat. For slender cores or regions with restricted heat access, a patent describes internal fluid circulation to deliver heat locally [64].

In complex molds, differences in heat-transfer distance can produce uneven thermal conditions even when the heat source is maintained at a constant temperature. Kuo et al. compared conventional and conformal heating channels in an optical-grade LSR mold and found that the conformal design improved heating response and mold-surface temperature uniformity. At a thermal-oil temperature of 180 °C, the simulated surface-temperature difference decreased from approximately 16.86 °C to 8.19 °C [65]. The comparison shows how adapting the channel layout to the cavity geometry can improve heat distribution, complementing control of the heat-source temperature.

Alternative heat sources offer further ways to modify thermal delivery. Related rubber-processing studies have examined induction-heating temperature fields in a vulcanizing machine [66] and silicone-rubber curing under high-intensity infrared radiation [67]. These approaches change how energy reaches the tooling or material, making heat penetration, temperature distribution, and curing time central considerations for their use in an LSR molding cycle. Figure 2 brings together the equipment functions described in Section 4.1, Section 4.2, Section 4.3 and Section 4.4, showing how metering, injection, nozzle cooling, and mold heating establish the material-delivery path and its thermal conditions.

### 4.5. Intelligent Monitoring

While equipment establishes the processing conditions, sensing reveals how the material and mold respond during operation. Monitoring records process conditions and their variations, while prediction uses measurements and models to estimate material states or quality outcomes. Optimization identifies processing settings for specified objectives, and closed-loop control updates operating variables within or between cycles using feedback. These tasks can be applied in combination yet play distinct roles when translating process information into equipment actions. Cavity pressure is particularly informative because its evolution reflects filling and holding, thermal expansion, and volume changes associated with crosslinking [68]. Weißer et al. identified characteristic pressure-curve changes during crosslinking and used signal differentiation to distinguish curing stages [69]. Chang et al. applied wavelet analysis to optical LSR molding, using noise reduction and feature extraction to examine velocity-to-pressure (V/P) switchover and relationships with transmittance, shrinkage, and residual stress [70]. Together, these studies show how pressure records can support both process interpretation and quality assessment when their features are related to the relevant molding stage.

The choice of sensing method also depends on access to the cavity. Limited installation space and demanding operating conditions can favor external measurements [71]. In LSR automotive-headlamp-lens molding, tie-bar strain measurements recorded clamping-force variations and were combined with a deep neural network and non-dominated sorting genetic algorithm III (NSGA-III) optimization. Based on the optimization results tabulated in the study, residual stress and clamping force decreased by 12.98% and 47.14%, respectively, relative to the initial settings [72]. In thermoplastic injection molding, multiple in-mold sensors have also been used for process and quality control [73]. These measurements provide information at different spatial scales: cavity pressure reflects local process responses, mold-temperature sensors characterize local thermal conditions, and tie-bar strain captures the overall machine–mold loading response. Their usefulness for material-state estimation depends on how the measured quantities are related to the states of interest. Among the studies reviewed, pressure-based interpretation of curing was supported by differential scanning calorimetry (DSC) measurements in a dedicated test device [69], while pressure features used for optical-quality assessment were evaluated against transmittance, shrinkage, and residual stress [70]. These two forms of validation address different questions: the former supports interpretation of reaction progress, whereas the latter establishes relationships between process features and final part quality. Mold-temperature measurements characterize the thermal conditions relevant to pressure-signal interpretation and provide a basis for evaluating heat-transfer predictions [65]. Together, complementary measurements and calibration appropriate to the formulation, sensor locations, and molding conditions support the interpretation of process signals for material-state estimation.

Turning these measurements into useful estimates involves choosing a model according to the quantity to be inferred. Physical models can incorporate curing parameters obtained from DSC [74], providing a description of reaction progress, whereas data-driven methods relate measured histories to control targets or quality outcomes [75,76,77,78]. Much of the latter work concerns thermoplastic injection molding and offers methodological guidance for LSR. Pressure-profile interpretation and model calibration address the relationship between a recorded signal and the process response [79,80]; dimensionality reduction and feature extraction instead determine which information from transient records is retained for prediction [81,82]. Part-weight prediction and control [83] and explainable modeling [84] bring these methods closer to production decisions, but address different endpoints: predicting a quality indicator, interpreting its associations with process features, and selecting a corrective action. For LSR, these tasks are connected by the need to distinguish filling, thermal expansion, and network development. Combining kinetic descriptions with signal analysis could help determine whether an observed deviation calls for an adjustment to material delivery or to the thermal and curing conditions.

Moving from prediction or optimization to closed-loop control requires an adjustment to be implemented and its effect to be evaluated through feedback. Thermoplastic injection-molding studies describe closed-loop retrofitting, feedback control, and cross-phase predictive cavity-pressure control [85,86,87], while industrial LSR reports describe within-cycle parameter adjustment [56]. These approaches connect measurements with intervention at different times within the molding sequence. Their relevance to LSR lies in matching the adjustment to the process response—for example, using pressure-related information to review injection settings and measured mold temperature to regulate heater power. Reviewing settings supports process decisions; closed-loop operation additionally uses a defined feedback rule to execute adjustments and subsequent measurements to guide further action. Process measurements indicate whether the controlled response approaches its target, while part measurements establish whether the adjustment improves the dimensions or properties of interest. Table 2 compares the measured quantities, interpretation targets, functional roles, and reported validation, clarifying how each approach contributes to monitoring, prediction, optimization, or feedback control.

**Figure 3:**
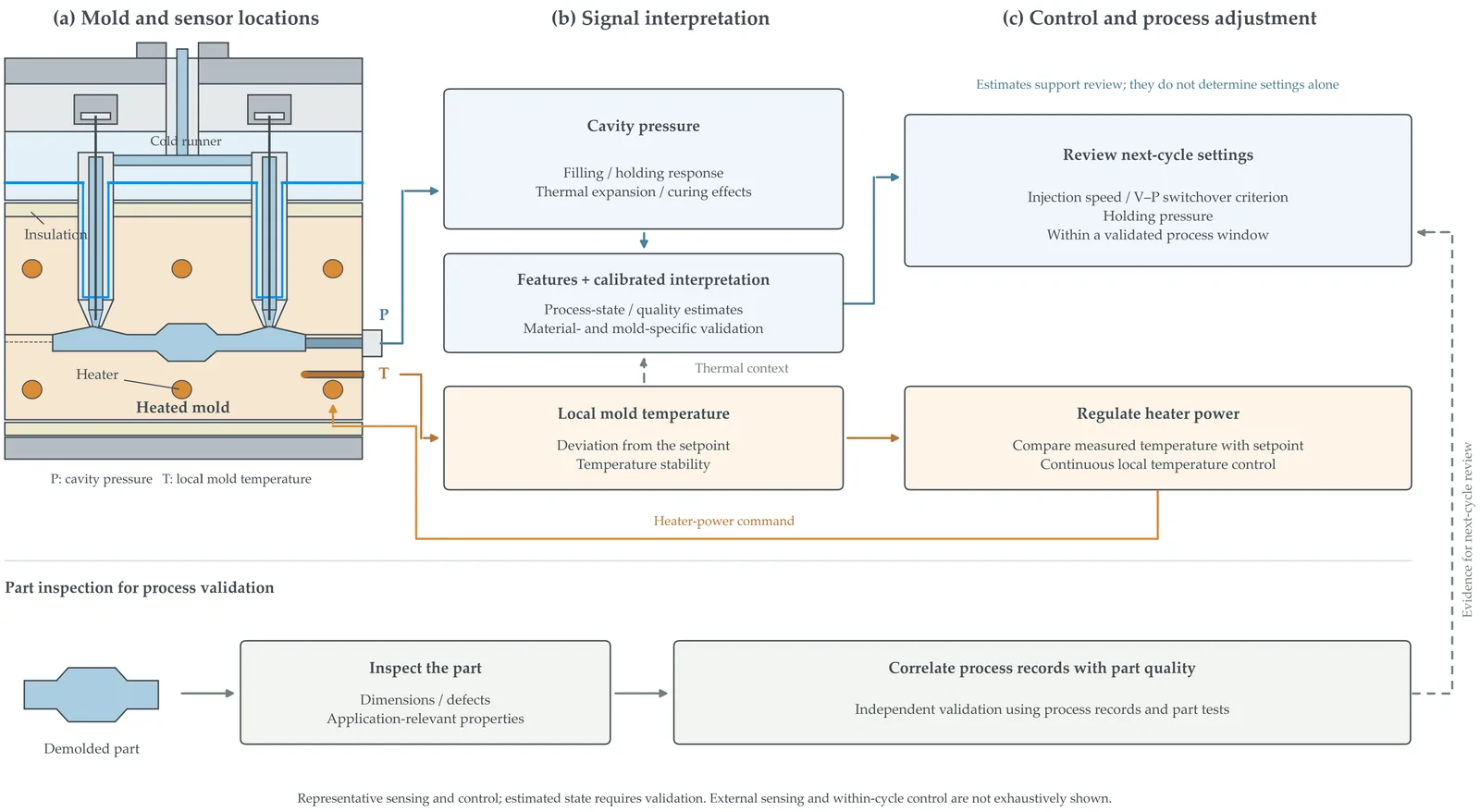
Process monitoring, signal interpretation, and control in LSR injection molding. (**a**) Representative locations for cavity-pressure (P) and local mold-temperature (T) measurements; (**b**) interpretation of measured signals through extracted features and calibrated relationships; and (**c**) review of next-cycle processing settings and regulation of heater power. Part inspection links process records with measured product quality and informs subsequent process review. Material-state estimates are interpreted for the formulation and mold considered. The diagram is conceptual.

The relative importance of a given control variable across the entire equipment chain depends on the quality outcome of interest. For dimensional accuracy, injection and packing conditions, velocity-to-pressure switchover, and mold temperature jointly shape the material flow, pressure and temperature histories, thereby affecting shrinkage and final dimensions [48,88]. For mechanical properties, accurate metering and homogeneous mixing of components define the composition of material entering the mold, while mold temperature and curing time regulate subsequent network evolution [32,41]. Component control and thermal management together link equipment operation to the mechanical response of cured articles [88]. For defect formation, injection conditions interact with runner geometry, venting and parting-surface sealing, governing whether the cavity can be fully filled, displaced air can be exhausted, and material can be effectively confined within the intended molding volume [10,47,89]. These relationships highlight the need to coordinate material delivery and mold operation when selecting settings for the required part quality. Cavity pressure records and temperature measurements provide complementary information for evaluating the resulting process response [65,69,70]. A key next step is to connect equipment adjustments to independently characterized material states and repeatable quality outcomes across batches and molds. Figure 3 summarizes this process via sensing, signal interpretation and process tuning, and uses part inspection results to inform process review for subsequent cycles.

## 5. Quality Formation and Defect Control

### 5.1. Dimensional Accuracy and Shrinkage

Dimensional accuracy reflects how closely a molded LSR part conforms to its intended geometry. Dimensional consistency reflects the combined effects of material delivery, pressure and temperature during filling and curing, and cooling after demolding. Using experimental design, Barbaroux et al. showed that mold temperature, holding pressure, runner configuration, and process-switchover conditions affect the mass, volume, dimensions, and mechanical properties of injection-molded silicone rings [88]. These interconnected responses make dimensional control a matter of coordinating the molding cycle rather than adjusting a single parameter.

Pressure also links processing conditions to the structure of the cured material. Barbaroux et al. used equilibrium solvent swelling to assess the crosslink density of parts molded under different temperature and pressure conditions [90], providing experimental evidence of how the network responds to molding conditions. Together with the dimensional measurements discussed above [88], these findings support considering pressure history and network development jointly when interpreting shrinkage. The two studies provide complementary evidence on network structure and final dimensions.

For optical components, this dimensional assessment also extends to internal stress and local shape. Chang et al. investigated LSR lens arrays using filling time, compound temperature, curing pressure, and curing time as optimization variables, with volumetric shrinkage and residual stress as quality indicators [91]. Treating these indicators together captures different consequences of the same processing history. Volumetric shrinkage describes the overall change in volume, while local dimensional measurements and warpage assessment reveal how that change is distributed within the part.

### 5.2. Surface Quality and Replication Fidelity

At a finer scale, dimensional control concerns the reproduction of individual mold-surface features. In microstructured LSR parts, these features can also provide the intended surface function. Hopmann et al. produced superhydrophobic LSR surfaces with conical microstructures through single-step injection molding [92], linking the formation of the molded texture directly to its wetting behavior.

Replication depends on both the feature geometry and the conditions under which the compound enters it. Lozano-Hernández et al. used hybrid polymer inserts to reproduce micro- and nanotextures in LSR, examining surface structures with characteristic dimensions of approximately 2–50 μm. Texture geometry and processing conditions jointly influenced replication fidelity and final wetting behavior [93]. For optical microstructures, Kuo and Lin reported approximately 91.5% replication of microgroove depth and 99.2% replication of groove width in LSR Fresnel-lens molding, with microgroove dimensional variations within approximately ±1 μm [94]. The difference between depth and width replication shows that individual features can respond unevenly during molding. Assessing the dimensions relevant to function consequently provides more useful information than a single overall replication value.

For optical parts, the quality of the replicated geometry is closely connected with the condition of the material beneath the surface. Chang et al. reported a negative correlation between residual stress and optical transmittance in LSR, while nonuniform volumetric shrinkage affected dimensional accuracy and optical performance [70]. Surface-profile measurements and residual-stress assessment thus describe complementary aspects of optical quality: one captures the reproduced geometry, and the other helps explain performance differences that geometry alone may not reveal.

### 5.3. Mechanical Properties and Network Uniformity

Beyond geometry and surface function, the cured network governs how the part carries load and accommodates deformation. Delebecq et al. examined relationships between network topology and dynamic mechanical behavior, compression set, tensile properties, elongation at break, and tear resistance [95]. Compression behavior was associated with molecular-chain mobility and filler–polymer interactions, whereas elongation at break depended on the molar mass between crosslinks and the distribution of elastic strands. Different mechanical tests therefore probe different aspects of the same network, making their results complementary when evaluating a molded component.

Changes in formulation can shift these responses by different amounts. Studies of nanoclay and carbon-black reinforcement illustrate the contribution of fillers to LSR performance [96,97]. Mayer et al. reported that compounds containing up to 40 wt.% recycled powder could be processed using conventional liquid injection molding (LIM) equipment. In the reported mechanical results, compression set remained nearly unchanged, while hardness decreased and tensile strength and elongation at break were reduced by approximately 16.6% and 12.6%, respectively [98]. This contrast makes the intended loading condition central to formulation assessment: retention of compression-set performance may be useful for one application, while reductions in tensile strength or extensibility may be more important for another.

Processing history further modifies the response established by formulation. Mechanical characterization of bulk Sylgard 184 provides information on PDMS behavior in that material system [99], while studies of injection-molded silicone address the additional influence of molding conditions. In particular, Barbaroux et al. showed that mold temperature, pressure, runner conditions, and process transitions can alter the mechanical properties of molded parts [88]. Evaluating these effects involves both the overall mechanical response and its spatial consistency. Bulk tests characterize the response of the specimen as a whole; assessing network uniformity additionally calls for measurements that resolve differences within the part.

### 5.4. Defect Formation and Control

The same interactions that shape dimensions and mechanical properties can also produce bubbles, voids, short shots, and flash when filling, venting, and curing conditions are poorly coordinated. Park et al. investigated process-parameter variation alongside cavity-pressure histories and mechanical response, including the influence of venting and curing conditions [47]. Relating the observed defect to the process record helps identify which stage of molding contributed to its formation and which adjustment is most likely to address it.

The balance between venting and sealing is particularly clear at the parting surface. Low-viscosity LSR can penetrate narrow gaps, yet closing these gaps without preserving an air-release path can increase air entrapment. Uhlmann et al. investigated mold-insert finishing and parting-surface design, using electrical-discharge dressing to produce closely fitting inserts and examining the role of EDM surface roughness in venting. They reported flash-free molding at low clamping forces in the studied setup [89]. This result highlights the contribution of surface fit and venting geometry to flash control, alongside the applied clamping force.

The importance of a deviation also depends on the function of the component. Optical LSR parts may exhibit shrinkage-related shape changes or residual stress even when their surfaces appear free of defects. Chang et al. therefore considered volumetric shrinkage and residual stress together in the multi-objective optimization of silicone-lens arrays [91]. For such components, defect assessment extends from visible imperfections to changes that affect optical performance. Table 3 summarizes the evidence linking formulation and processing to part quality.

Internal defects add another dimension to quality evaluation, as their location and extent cannot necessarily be determined by surface inspection alone. Relevant studies on silicone rubber systems have demonstrated several complementary detection methods. X-ray micro-computed tomography (micro-CT) has been applied to reconstruct pores and cracks in silicone rubber insulators, delivering three-dimensional information on defect morphology and spatial distribution [100]. For interfacial defects, ultrasonic pulse-echo imaging can reconstruct debonded regions in room-temperature vulcanized silicone rubber coatings and estimate their dimensions [101]. Terahertz imaging offers an alternative approach for subsurface defect detection: Choi employed a solid-immersion-lens-based system to resolve near-surface voids of approximately 100 μm in both depth and lateral dimension within silicone rubber [102]. Table 4 compares these methods in terms of the material systems examined, target defects, information obtained, and considerations for method selection. Relating the inspection results to filling, venting, and curing records can help assess whether process adjustments have reduced the defects of interest.

**Figure 4:**
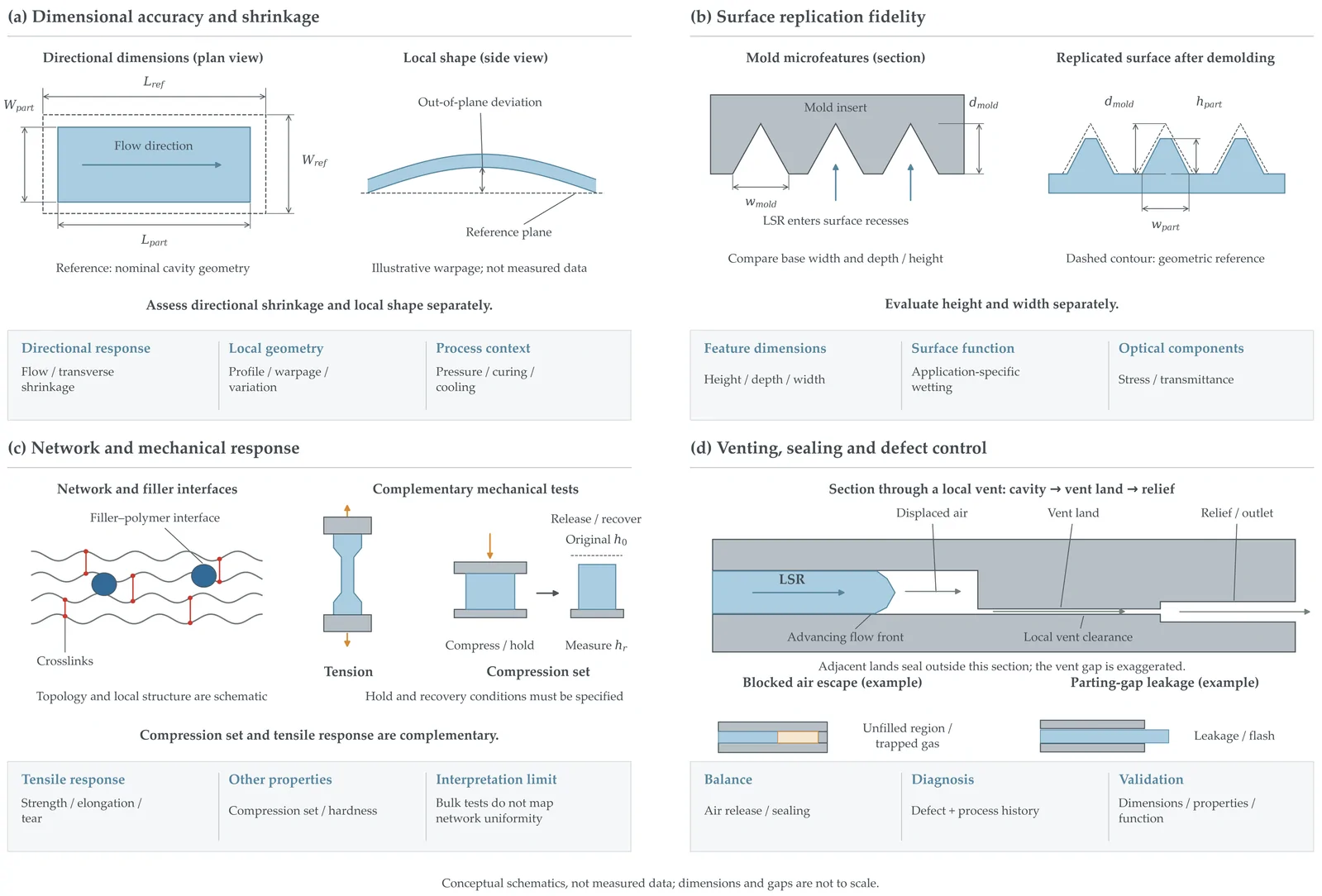
Quality formation and assessment of injection-molded LSR parts. (**a**) Directional shrinkage and local shape deviation; (**b**) replication of mold-surface features; (**c**) schematic network and filler structure together with complementary tensile and compression-set assessments; and (**d**) local venting and examples of defects associated with restricted air escape and parting-gap leakage. Dashed contours indicate geometric references where shown. Network structures, deformations, and vent clearances are illustrative rather than measured and are not to scale.

Therefore, corrective adjustments must balance filling, curing, and dimensional requirements. Increasing injection pressure may facilitate filling in regions with high flow resistance, but can also promote material penetration into parting gaps, making its effectiveness dependent on sealing and venting conditions [10,89]. Similarly, reducing vent clearance can restrict flash but may impede air removal. Thermal adjustments introduce another trade-off: a higher mold temperature can accelerate curing and shorten the required curing stage, while faster network development may restrict local filling and changes in thermal history affect shrinkage [27,41,48]. Cycle-time reduction is beneficial when the part retains sufficient integrity for demolding and meets dimensional and mechanical requirements. Process selection should balance these outcomes according to the intended function of the component, rather than optimize filling completeness, flash suppression, or cycle time separately. Figure 4 brings together the dimensional, surface, mechanical, and venting-related outcomes that need to be considered when selecting processing conditions.

## 6. Industrial Applications and Functional Integration

### 6.1. Medical and Biomedical Devices

In medical and biomedical devices, dimensional reproducibility is accompanied by requirements for material purity, biological response, and stability during use. Studies of PDMS microfluidic cell culture show how absorption by the material can influence the biological environment [103], while the broader biomaterials literature relates PDMS properties to its applications [104]. The material–tissue or material–fluid interface is therefore an important part of device design. Studies of silicone hydrophilization [105], breast-implant surface modification [106], and comparative in vitro immune-cell responses to surface-modified LSR and thermoplastic polyurethanes [107] examine how modifying this interface changes its interaction with the biological environment.

For devices that combine rigid support with compliant contact, multicomponent molding can integrate these functions while reducing subsequent assembly. Nikousaleh et al. investigated medical-grade acrylonitrile–butadiene–styrene (ABS)/LSR injection molding, using dynamic mold-temperature control and surface pretreatment to accommodate the thermal limitations of ABS and improve its otherwise insufficient adhesion to LSR. They also evaluated interfacial stability after sterilization [108]. This extends interface assessment from bond formation during molding to performance during device use. Repeated cleaning, disinfection, and sterilization have been shown to alter surface condition, hardness, crack growth, and silicone–metal interfacial integrity in medical-grade silicone systems [109], making resistance to these treatments part of the material and process selection.

Injection molding also allows localized surface structures to be incorporated into three-dimensional components. Zhang et al. replicated micropillars on LSR parts for potential tissue-anchoring interfaces in implantable electrodes and evaluated replication over 1000 injection cycles [110]. Their work addresses the manufacturing consistency of a geometry intended for biological interaction. Surface modification provides a complementary way to influence that interaction. A review of PDMS-based functional materials distinguishes bulk modification from surface functionalization [111], while Lei et al. combined microgroove patterning with carbon-ion implantation to improve the biological response of silicone surfaces [112]. Together, these approaches connect molded geometry with surface chemistry in the design of the tissue-contacting interface.

Microfluidic devices bring these material and surface considerations into structures that must also handle fluids reproducibly. Established PDMS fabrication methods often use laboratory-scale casting [113,114]. Filion et al. instead used industrial LSR injection molding for mass fabrication of PDMS microfluidic devices, reporting reduced batch-to-batch variation in several bulk and surface properties compared with conventional laboratory processing. The devices also supported three-dimensional tumor spheroid and tissue explant cultures [115]. This study connects manufacturing consistency with a demonstrated biological application, extending the role of injection molding from reproducing channel geometry to producing devices with more consistent material characteristics.

### 6.2. Optical and Automotive Lighting Components

In optical applications, molded geometry works together with material transparency and thermal stability to determine performance. Optical-grade LSR is used in light-emitting diode (LED) encapsulation, Fresnel lenses, free-form optics, and automotive lighting, where these properties support complex optical designs [94,116,117,118]. Fresnel-lens studies show that injection speed, pressure, mold temperature, and time influence microfeature replication [94]. The processing conditions therefore shape the optical profile while also establishing the curing and stress state of the material.

The same molding operation can combine optical formation with encapsulation. Hopmann and Röbig placed an LED circuit board in the mold and overmolded it with transparent LSR, integrating primary optical encapsulation and the optical head within one component [117]. This reduces the number of separate parts and assembly operations, while bringing insert positioning, encapsulation integrity, and optical geometry into the same manufacturing step. Process selection consequently involves both reproducing the optical surface and maintaining the position and integrity of the embedded assembly.

The significance of these manufacturing requirements becomes clearer at the lighting-system level. Hu et al. investigated LSR light guides for compact automotive headlamps [118], in which component geometry must be considered in relation to the intended light distribution and system configuration. Dimensional and material assessments thus support a functional evaluation of the assembled optical system. For overmolded components, this evaluation also captures the combined effects of the LSR geometry and its integration with the insert.

### 6.3. Multi-Material and Integrated Components

The integration illustrated by LED overmolding extends to components that combine structural and compliant functions. Thermoplastic/LSR multicomponent molding brings rigid support together with flexibility, sealing, damping, or tactile response within one part [119]. The interface transfers these functions between the constituent materials, making adhesion central to the performance of the integrated component. Surface condition and thermal history influence bonding during overmolding [120,121], so material pairing and process selection are closely connected.

Ou et al. examined this connection by combining a coupled thermal–rheological–curing model with polyamide/LSR overmolding experiments and interfacial adhesion evaluation [122]. Their approach places bond formation within the spatially varying thermal conditions experienced by the substrate and LSR during molding. Surface treatments modify another part of the same interface. Seitz et al. compared atmospheric-pressure plasma and Pyrosil flame treatment for self-adhesive silicone rubber/thermoplastic joints [123], while Rüppel et al. applied silicatization to polypropylene before LSR overmolding and evaluated adhesion after storage [124]. These studies address different stages of joint performance: the coupled molding analysis relates processing conditions to adhesion, the treatment comparison evaluates surface preparation, and storage testing examines whether the resulting adhesion persists. Their results are most useful for process selection when the material pair, treatment conditions, and test endpoint are considered together.

Treatment selection also depends on the specific material pair. Hartung and Heim found that ultraviolet C (UVC)/ozone treatment could activate polycarbonate surfaces and promote LSR adhesion, whereas ozone-free UVC treatment produced lower peel strength and a pronounced dependence on LSR grade [125]. The contrast shows why treatments cannot be ranked independently of the substrate and LSR grade. A treatment that gives high initial peel strength addresses bond formation, while storage or exposure tests address adhesion retention; transparent substrates introduce the additional requirement of preserving optical performance. Selecting a treatment therefore involves balancing these outcomes for the intended material pair and service conditions.

### 6.4. Functional Surfaces, Wearable Interfaces, and Soft Robotics

Functional integration can also be achieved within the silicone component itself through designed surface features, conductive modification, and internal cavities. In wearable interfaces and soft actuators, these features work with material compliance to establish contact or generate movement. Reviews of soft-robot manufacturing [126] and silicone materials for soft actuators [127] describe several fabrication routes, with injection molding providing one means of reproducing the required structures.

For wearable electrodes, surface geometry influences how the conductive material contacts the skin. Kaitainen et al. investigated conductive LSR dry bioelectrodes with micropillar structures and silver coatings, examining their relationship with contact impedance [128]. Millimetre-scale conductive silicone protrusions have also been studied for electrical contact [129]. These examples connect geometric design with electrical performance: feature dimensions describe the manufactured surface, while impedance measurements assess how that surface functions as a contact interface.

Internal geometry serves a different purpose in soft actuators, where hollow structures accommodate the pressure or other actuation input that produces deformation. Bryantono et al. used lost-core injection molding to manufacture hollow soft grippers from low-viscosity silicone, combining injection with a removable water-soluble sacrificial core [130]. The core defined the internal geometry during molding and was subsequently removed to leave the cavities. Ishikawa et al. proposed an in-vacuum silicone-rubber forming process for soft robots [131], offering a different route for producing complex structures. Comparing such routes involves how internal features are formed, supported during processing, and made accessible after fabrication.

**Figure 5:**
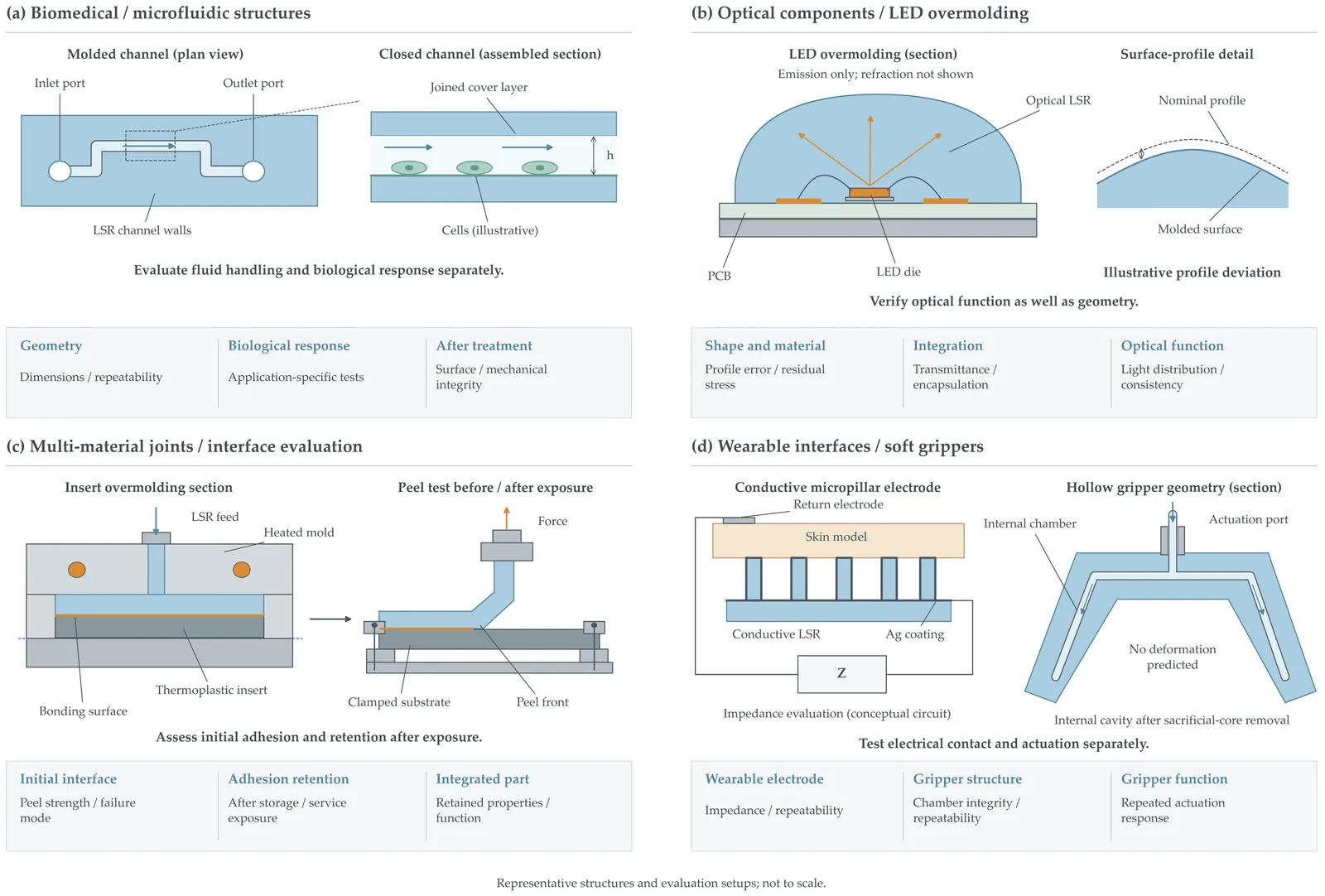
Representative functional structures and application-specific assessment of LSR components. (**a**) Microfluidic channels and an assembled channel section; (**b**) LED overmolding and optical-surface profile assessment; (**c**) insert overmolding and interfacial peel assessment; and (**d**) conductive micropillar interfaces and hollow soft-gripper structures. The examples distinguish geometric assessment from biological, optical, interfacial, electrical, and actuation performance. Test arrangements are conceptual; the electrode circuit does not isolate individual impedance contributions, and the gripper schematic does not predict deformation.

Across these applications, repeatable material preparation, controlled curing, and geometric consistency form a shared manufacturing foundation, while the intended function guides the emphasis placed on different process elements. For biomedical devices, material interactions with fluids or tissues, together with resistance to sterilization where applicable, guide formulation, surface treatment, and interface design. Optical components place greater emphasis on surface-profile replication, internal stress, and optical transmittance, linking filling and thermal management to optical performance. Multi-material parts rely on adhesion and its retention during service, making substrate pretreatment and thermal compatibility central to process selection. Wearable electrodes and soft grippers extend these requirements to electrical contact and deformation behavior, respectively; their surface features and internal cavities must therefore be assessed in relation to the functions they enable. Table 5 connects manufacturing routes and reported processing conditions with application-relevant tests, while Figure 5 illustrates the corresponding structures and assessment methods. A transferable principle is to coordinate material delivery and curing with the functional structures being produced; specific processing settings and acceptance criteria depend on the material system, geometry, and intended use.

## 7. Challenges and Future Perspectives

### 7.1. From Process Monitoring to Material-State-Based Control

A key direction for LSR process monitoring is to use measured signals to guide equipment adjustments. For LSR, a central challenge is relating those signals to material state as formulations, molds, and machines change. Comparisons of viscosity-characterization methods [132], together with the differences in injection-pressure predictions reported by Azevedo et al. [28], show how the material description influences the predicted process response. State estimation therefore begins with rheological characterization that represents the shear and thermal conditions experienced during molding.

Building on this description, online measurements can be used to estimate quantities that guide equipment adjustment, such as filling progression or degree of cure. Machine-learning control of recycled-polypropylene injection molding provides a methodological example of linking process data to control [75]. In LSR, the additional contribution of crosslinking makes the combination of data-driven methods with rheological and kinetic models particularly relevant. Physical descriptions can help distinguish flow-related signal changes from network development, while production data capture variations that are difficult to represent explicitly. Independent measurements of the material state provide an anchor for interpreting signals with overlapping physical contributions.

Digital-twin studies in polymer injection molding offer a framework for updating these models with production data [133,134]. For LSR, this creates an opportunity to incorporate batch variation and changing mold conditions into the relationship between estimated state and equipment response. Injection speed and V/P switchover act primarily on material delivery, whereas mold temperature and curing time regulate thermal exposure and network development. A feedback strategy needs to reflect these different response times and the stages at which each adjustment remains effective.

Transferring these methods to different production conditions requires consideration of changes in both material responses and measurement–control relationships. Changes in formulation alter rheological behavior, curing kinetics, and thermal properties; accordingly, pressure or temperature features associated with a given material state may vary between formulations. Variations in mold geometry, heat-transfer pathways, and sensor locations modify the conditions represented by recorded signals, while differences in machine response and control implementation affect how control commands alter the actual process. Transfer therefore involves more than applying the same predictive model to new data. It requires aligning material descriptions and signal interpretation with the new conditions and adapting control actions to the responses of the target machine and mold. One viable route is to retain transferable physical relationships while recalibrating formulation-dependent parameters, geometry- and sensor-location-dependent signal features, and equipment-response models. Evaluating state-estimation accuracy together with the achieved reduction in part-quality variation can help determine whether such adaptation improves manufacturing consistency.

### 7.2. High-Efficiency Thermal Management

Among the available control actions, thermal management directly influences the rate and spatial development of curing. Kuo et al. developed an LSR fisheye-lens mold with conformal thermal-fluid channels and reported improved vulcanization efficiency [135]. Adapting heat-transfer paths to the cavity geometry thus provides a basis for further control over where and when heat is supplied.

This spatial control becomes particularly relevant in parts with varying thickness. Even with a uniform mold-surface temperature, different regions can follow different internal temperature histories and reach the required curing state at different times. Combining thermal prediction and curing-state estimation with zoned heating could allow local heat input to follow these differences. The resulting strategy would coordinate the curing of slower-heating regions with that of regions already approaching the required state, linking heater operation more closely to the development of the part.

Rapid-response methods such as infrared and induction heating offer additional ways to adjust heat delivery. Their use in industrial LSR injection molding remains an area for development, with heat penetration and coupling to the tooling or material as important as the heating rate itself. Comparisons under consistent mold and material conditions would clarify how the heat source and its spatial arrangement each contribute to cycle time and temperature distribution.

The practical objective is to reduce the time and energy required per acceptable part. Evaluating heating strategies through cycle time, energy consumption, production yield, and property consistency would capture the combined effects of faster curing and the quality achieved.

### 7.3. Resource-Efficient and Sustainable Manufacturing

The same focus on acceptable output connects thermal efficiency with material use. Cold runners, accurate dosing, and defect prevention reduce losses at the source, while reuse and recovery address the scrap that remains. Broader reviews of silicone recycling distinguish mechanical reuse from chemical recovery [136]. These routes enter the manufacturing chain at different points: mechanical reuse introduces cured particles into a new compound, whereas chemical recovery seeks feedstocks for subsequent material preparation.

In mechanical reuse, the retained particles become part of the formulation and influence both processing and final properties. Liedl et al. reintroduced ground LSR at 10–30 wt.% while varying particle size and hardness; the mixtures were formed into plates by hot pressing and subsequently post-cured [137]. Property retention depended on the material system: losses were generally smaller for the Shore A 50 and 70 materials, whereas the Shore A 30 formulation containing 10 wt.% of 0.2 mm particles showed reductions in tensile strength and elongation at break approaching one half. Compared with the conventional injection-molding study discussed in Section 5.3, which reported similar compression set but lower tensile properties [98], this work adds evidence on the effects of matrix hardness and particle characteristics under a different preparation route. The studies therefore support selecting recycled content together with the receiving formulation, processing route, and target property, rather than treating recycled content alone as a predictor of performance. Work on ground silicone vulcanizate as filler [138], recyclate in high-consistency silicone rubber [139], and cryo-milled silicone waste in silicone formulations for pressure sensors [140] further illustrates how the receiving matrix and intended function shape the reuse route. For injection-grade LSR, particle dispersion and compound flow must be considered alongside the mechanical requirements of the next product.

Chemical recovery moves the point of reuse from cured particles to recovered molecular feedstocks. Studies of silicone degradation and chemical recycling [141,142,143,144,145,146,147], together with alcoholysis of high-consistency silicone rubber [148], provide approaches to obtaining such materials. Dynamic-network silicone vitrimer composites address reprocessability through a different strategy, changing the network design itself [149]. For conventional addition-cure LSR, the route from recovered feedstock to an injection-moldable formulation remains a key development task. It brings purification and polymer preparation into direct contact with formulation requirements: suitable viscosity, reliable platinum-catalyzed curing, material purity, and retained long-term performance.

**Figure 6:**
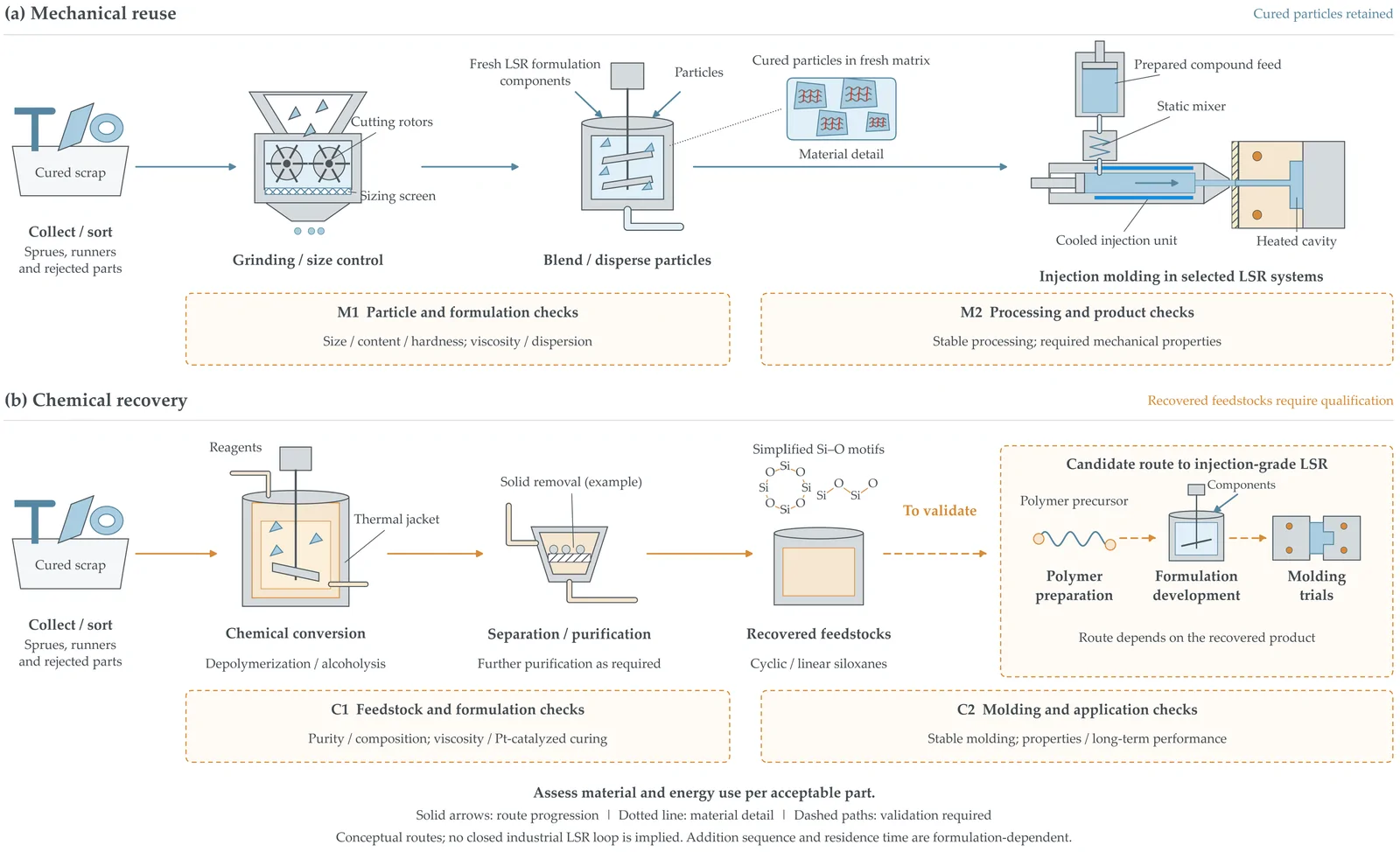
Mechanical reuse, chemical recovery, and assessment of silicone waste for reprocessing. (**a**) Size reduction and incorporation of cured particles into a fresh formulation, followed by processing and product checks; and (**b**) chemical conversion, separation, and feedstock recovery, followed by a candidate route toward injection-grade LSR. Solid arrows indicate route progression, dotted lines connect material details, and dashed paths indicate steps requiring validation. Chemical structures represent simplified siloxane motifs. The routes are conceptual; a closed industrial recycling loop for conventional addition-cure LSR is not implied.

Following the recovered material through these stages would make recycling studies more informative for manufacturing. Production scrap such as sprues and runners offers a potential feedstock [137], but collection, treatment, and reincorporation also consume resources. Assessing material and energy use per acceptable part would connect recovery efficiency with the processing and performance of the next product. Figure 6 organizes these questions into two routes, pairing particle and formulation checks with processing and product assessment for mechanical reuse, and feedstock qualification with formulation development and molding trials for chemical recovery.

### 7.4. Multi-Material and Hybrid Manufacturing

Resource efficiency also benefits from integrated components that retain their function throughout use. In multi-material LSR molding, this places particular importance on the durability of the joint. Mayer et al. investigated thermoplastic–LSR surface treatments and reported improvements in surface energy and peel performance using atmospheric-pressure plasma and silicating treatments [150]. The next question is how these initial improvements persist during repeated deformation, thermal aging, sterilization, or chemical exposure, particularly when several of these conditions occur together.

Answering this question involves following the interface from substrate preparation through second-shot molding and service. Surface chemistry and substrate thermal history establish the conditions for bonding, while LSR curing and residual stress contribute to the joint that develops. Relating these preparation conditions to changes in failure mode and retained adhesion would help identify whether durability is governed primarily by the interface, the adjacent materials, or their interaction. This would support selection of surface treatments and molding conditions according to the demands of the application.

Hybrid manufacturing extends integration from material combinations to combinations of fabrication methods. Ultraviolet follow-curing of silicone [151] and printed silicone pneumatic actuators [152] offer approaches to forming silicone structures outside conventional mold filling, while sacrificial molds provide a route to shaping otherwise difficult geometries [153]. These capabilities suggest opportunities to complement LSR injection molding with printed inserts, localized functional structures, or removable cores for internal channels. The manufacturing benefit depends on how these elements are positioned, withstand the molding cycle, and are subsequently removed or bonded. Considering these operations together with molding would reveal their contribution to repeatability and total production time.

Geometric freedom is most useful when it enables a function that can be reproduced reliably. Repeated fabrication and service-relevant testing can connect the added design possibilities of hybrid processes with interface durability, dimensional consistency, and device performance. Across the research directions discussed in this chapter, the common task is to link a technical advance to a measurable production outcome: more consistent quality, lower resource use per acceptable part, or longer retention of function.

## 8. Conclusions

Precision LSR injection molding relies on the coordination of material flow, heat transfer, and crosslinking. Formulation establishes the rheological and curing responses of the compound, while material delivery, injection conditions, and mold thermal management jointly shape its processing history. The reviewed evidence links these conditions to shrinkage, surface replication, mechanical properties, and defects. Their effects are coupled: conditions favorable for filling may also alter flash formation, curing time, and dimensional response. Process selection therefore requires balancing competing quality objectives according to the material, geometry, and intended application.

This review connects equipment functions with material-state evolution and measurable quality outcomes, distinguishing experimental observations from model-based interpretations. Pressure and temperature records, material characterization, and part testing provide complementary evidence but address different questions. Experiments establish changes in process response and part performance, while models help interpret internal temperature and cure distributions. Further work is needed to independently validate the intermediate material states linking equipment adjustments to quality improvements, particularly across different formulations, molds, and machines.

Future research should build on these relationships to develop state-estimation methods that guide effective feedback and reduce part-to-part variation. Thermal management strategies should be evaluated in terms of cure distribution, product quality, and energy consumption per acceptable part. Reuse and recovery routes need to be integrated with formulation development and reprocessing, while multi-material and hybrid manufacturing depend on reproducible structures and retained interfacial or device performance. These priorities connect advances in materials and equipment with consistent, resource-efficient production.

## Figures and Tables

**Table 1:** Comparison of the scope and analytical emphasis of related reviews and the present review.

Comparison Dimension	Xing et al. [1]	Huang and Chen [6]	Bont et al. [10]	Present Review
Scope	Heat-resistant addition-cure LSR; component–property relationships	Silicone-rubber types, properties, manufacturing, and applications	LSR injection-molding variables and process modeling	Equipment–material–quality relationships in LSR injection molding
Processing stages	Formulation and network design	Curing and conventional/emerging fabrication routes	Material handling, injection, holding, curing, and post-processing	Metering and mixing through filling, curing, and part assessment
Equipment emphasis	Material-component design	Manufacturing-route overview	Injection equipment, tooling, and simulation	Dosing, injection units, cold runners, thermal management, and sensing
Quality indicators	Thermal stability and degradation resistance	Mechanical, thermal, electrical, and application-related properties	Shrinkage, defects, bonding, and process-prediction accuracy	Dimensions, replication, mechanical response, defects, and functional performance
Control emphasis	Formulation-based property tailoring	Material and manufacturing choices for function	Parameter selection, simulation, and process feedback	Monitoring, state estimation, optimization, and feedback linked to quality evidence
Scope boundaries and remaining questions	Material-centered; molding control is not the organizing theme	Broad coverage across silicone systems	Identifies gaps in characterization, venting, and bonding prediction	Examines validation and transferability; does not establish a universal operating window

Entries summarize the principal emphasis and scope boundaries of each review; they do not imply the complete absence of related topics.

**Table 2:** Representative process measurements, interpretation targets, and validation relevant to LSR injection molding.

Study and System	Directly Measured Quantity	Task and Interpretation Target	Validation	Key Limitation
Kuo et al. (2023) [65] LSR mold	Mold-surface temperature measured by thermography and thermocouples	Monitoring and model assessment: mold-temperature distribution and heating-layout evaluation	Comparison of measured and simulated temperatures	Characterizes mold thermal conditions; cure state requires a material and heat-transfer description
Weißer et al. [69] LSR	Pressure in a dedicated test device and mold cavity	Monitoring and interpretation: cure-related pressure feature	DSC in test device; molding simulation	Supports cure-related interpretation under the investigated formulation and thermal conditions
Chang et al. [70] Optical LSR	Cavity-pressure history	Signal analysis and optimization: pressure features and optical-quality assessment	Transmittance, shrinkage and residual stress	Connects pressure features with part quality rather than directly measuring network development
Chang et al. [72] LSR lens	Tie-bar elongation	Prediction and optimization: machine–mold response and parameter selection	Model-based optimization of clamping force and residual stress; transmittance tests on molded lenses	Provides a global response whose relation to local material state depends on the mold and loading conditions
Párizs et al. [73] ABS	Pressure sensors behind ejector pins	Feedback action: pressure-triggered V/P switchover	Pressure records and part mass	Demonstrates a filling-control approach in thermoplastics; does not characterize LSR curing

LSR, liquid silicone rubber; DSC, differential scanning calorimetry; V/P, velocity-to-pressure; ABS, acrylonitrile–butadiene–styrene; NSGA-III, non-dominated sorting genetic algorithm III. Interpretation and optimization do not alone demonstrate closed-loop control. ABS evidence is indirect for LSR.

**Table 3:** Representative links between LSR formulation, processing, and part quality.

Study	System/Variable	Evaluation	Main Finding	Key Limitation
Mayer et al. [32]	Optical LSR; A/B ratio	Thermal, rheological and mechanical tests	Mixing ratio alters reaction behavior and final properties	Formulation-specific
Bont and Johnston [48]	Thin LSR parts; filling and thermal conditions	Dimensions, simulation and DMA	Directional shrinkage varies with processing	Geometry- and process-dependent
Barbaroux et al. [88]	Silicone rings; molding conditions	Experimental design; dimensions and mechanical tests	Temperature, pressure and runner conditions affect dimensions and properties	Specific material and ring geometry
Barbaroux et al. [90]	LSR; cavity pressure	Equilibrium solvent swelling	Cavity pressure affects crosslink density	Temperature- and formulation-dependent
Kuo and Lin [94]	LSR Fresnel lens; microfeature replication	Feature-depth and width measurements	Part replication: 91.5% depth; 99.2% width	Geometry alone does not establish optical function
Mayer et al. [98]	LSR with cured-silicone powder	Molding and mechanical tests	Up to 40 wt% processed; similar compression set, lower tensile properties	Property retention depends on the metric
Uhlmann et al. [89]	LSR; insert fit and venting surfaces	Molding trials; flash assessment	Flash-free molding at low clamping force in the studied setup	Tool- and process-specific

LSR, liquid silicone rubber; DMA, dynamic mechanical analysis. Results refer to the materials, geometries, and test conditions investigated in each study.

**Table 4:** Complementary methods for internal defect assessment in silicone-rubber systems.

Method	Material System and Target defects	Information Obtained	Considerations for Method Selection
X-ray micro-CT [100]	Silicone-rubber insulator samples; pores and cracks	Three-dimensional defect morphology, size, distribution, and porosity	Match the imaging resolution and inspected volume to the target defect size and specimen geometry
Ultrasonic pulse-echo imaging [101]	RTV silicone-rubber coatings; interfacial debonding	Reconstructed debonding morphology and estimated defect dimensions	Select the probe and acoustic coupling for the coating geometry and propagation conditions
Solid immersion lens-based terahertz imaging [102]	Silicone-rubber insulation; subsurface voids and metallic inclusions	Depth-resolved defect images; near-surface voids of approximately 100 μm in both depth and lateral dimensions	Consider defect depth and optical access; the reported fine-scale detection used a solid immersion lens

Micro-CT, micro-computed tomography; RTV, room-temperature-vulcanized. Material systems and detected defects refer to the cited studies.

**Table 5:** Manufacturing routes, reported processing conditions, and quality evaluation methods for representative LSR and related silicone applications.

Application and Representative Study	Material/Component	Manufacturing Route	Reported Processing Conditions	Finished-Part Evaluation
Medical hard–soft components; Nikousaleh et al. [108]	ABS/LSR specimens	Multicomponent injection molding with dynamic mold-temperature control and surface pretreatment	UVC treatment: 20 s; alternative plasma treatment: 18 mm nozzle distance and 0.05 m/s traverse speed; Pyrosil treatment: 20 mm distance and 0.4 m/s traverse speed	Peel resistance and failure mode; adhesion after artificial aging and sterilization
Biomedical microfluidics; Filion et al. [115]	Injection-moldable PDMS microfluidic devices	Industrial LSR injection molding with vacuum-assisted filling	A/B ratio: 1:1; reported injection pressure: 10–1000 bar; mold temperature: 150–200 °C; production cycle approximately 1 min	Young’s modulus, contact angle, oxygen permeability, small-molecule absorption, and 3D spheroid/explant culture
Automotive optics; Chang et al. [72]	LSR headlamp lens arrays	Injection molding with simulation-based process optimization and product evaluation	Optimization ranges: material temperature 20–35 °C; pressure parameter termed “curing pressure” in the source, 30–60 MPa; time parameter termed “curing time”, 5–10 s	Optical transmittance testing; model-based residual-stress assessment
Multi-material integration; Hartung and Heim [125]	PC/self-adhesive LSR specimens	UVC-treated PC followed by LSR overmolding	PC cylinder/mold temperatures: 300/100 °C; LSR cavity temperature: 140 °C; heating time: 30 s	Peel testing at 100 mm/min and failure-mode classification
Soft robotics; Ishikawa et al. [131]	Room-temperature-curing silicone structures and actuators	In-vacuum pouring/forming	Silicone viscosities: 3–20 Pa·s; room-temperature curing; 2 min rest after pouring in the cylinder-forming comparison	Filling completeness and bubbles/voids; actuator deformation measurements

Conditions refer to the cited studies. Ranges, fixed settings, and optimization variables are identified separately. The in-vacuum process is included as a related manufacturing route discussed in Section 6.4.

## Data Availability

No new data were created or analyzed in this study. Data sharing is not applicable to this article.

## Abbreviations

The following abbreviations are used in this manuscript:

3D	Three-dimensional
ABS	Acrylonitrile–butadiene–styrene
DMA	Dynamic mechanical analysis
DSC	Differential scanning calorimetry
EDM	Electrical discharge machining
LED	Light-emitting diode
LIM	Liquid injection molding
LSR	Liquid silicone rubber
micro-CT	X-ray micro-computed tomography
NSGA-III	Non-dominated sorting genetic algorithm III
OPC UA	Open Platform Communications Unified Architecture
PC	Polycarbonate
PCB	Printed circuit board
PDMS	Polydimethylsiloxane
RTV	Room-temperature vulcanized
UVC	Ultraviolet C
V/P	Velocity-to-pressure
